# Exposure to residential traffic and trajectories of unhealthy ageing: results from a nationally-representative cohort of older adults

**DOI:** 10.1186/s12940-024-01057-3

**Published:** 2024-02-01

**Authors:** Sergio Gómez del Río, Elena Plans-Beriso, Rebeca Ramis, Rosario Ortolá, Roberto Pastor, Mercedes Sotos-Prieto, Adela Castelló, Rocío Olmedo Requena, José Juan Jiménez Moleón, Borja María Fernández Félix, Alfonso Muriel, Marta Miret, Jose Luis Ayuso Mateos, Yoon-Hyeong Choi, Fernando Rodríguez-Artalejo, Pablo Fernández-Navarro, Esther García-Esquinas

**Affiliations:** 1 Department of Preventive Medicine, Hospital Central de la Cruz Roja San José y Santa Adela, Madrid, Spain; 2 Department of Chronic Diseases, National Center for Epidemiology, Carlos III Institute of Health, Madrid, Spain; 3 Consortium for Biomedical Research in Epidemiology, Public Health (CIBER en Epidemiología y Salud Pública - CIBERESP), Madrid, Spain; 4 Department of Preventive Medicine and Public Health, School of Medicine, Universidad Autónoma de Madrid, Madrid, Spain; 5 Department of Environmental Health, Harvard T.H. Chan School of Public Health, Boston, MA United States; 6 IMDEA-Food Institute (CEI UAM+CSIC), Madrid, Spain; 7 Department of Preventive Medicine and Public Health, School of Medicine, University of Granada, Granada, Spain; 8 Instituto de Investigación Biosanitaria ibs. GRANADA, Granada, Spain; 9 Clinical Biostatistics Unit, Hospital Ramón y Cajal (IRYCIS), Madrid, Spain; 10 Department of Nursery and Physiotherapy, Universidad de Alcalá, Madrid, Spain; 11 Department of Psychiatry, Universidad Autónoma de Madrid, Madrid, Spain; 12 Consortium for Biomedical Research in Mental Health (CIBER en Salud Mental - CIBERSAM), Madrid, Spain; 13 Department of Psychiatry, Hospital Universitario de La Princesa, Madrid, Spain; 14 School of Health and Environmental Science, College of Health Science, Korea University, Seoul, Korea

**Keywords:** Traffic pollution, Unhealthy ageing, Frailty

## Abstract

**Background:**

Traffic exposure has been associated with biomarkers of increased biological ageing, age-related chronic morbidities, and increased respiratory, cardiovascular, and all-cause mortality. Whether it is associated with functional impairments and unhealthy ageing trajectories is unknown.

**Methods:**

Nationally representative population-based cohort with 3,126 community-dwelling individuals aged ≥60 years who contributed 8,291 biannual visits over a 10 year period. Unhealthy ageing was estimated with a deficit accumulation index (DAI) based on the number and severity of 52 health deficits, including 22 objectively-measured impairments in physical and cognitive functioning. Differences in DAI at each follow-up across quintiles of residential traffic density (RTD) at 50 and 100 meters, and closest distance to a petrol station, were estimated using flexible marginal structural models with inverse probability of censoring weights. Models were adjusted for sociodemographic and time-varying lifestyle factors, social deprivation index at the census tract and residential exposure to natural spaces.

**Results:**

At baseline, the mean (SD) age and DAI score of the participants were 69.0 (6.6) years and 17.02 (11.0) %, and 54.0% were women. The median (IQR) RTD at 50 and 100 meters were 77 (31-467) and 509 (182-1802) vehicles/day, and the mean (SD) distance to the nearest petrol station of 962 (1317) meters. The average increase in DAI (95%CI) for participants in quintiles Q2-Q5 (vs Q1) of RTD at 50 meters was of 1.51 (0.50, 2.53), 0.98 (-0.05, 2.01), 2.20 (1.18, 3.21) and 1.98 (0.90, 3.05), respectively. Consistent findings were observed at 100 meters. By domains, most of the deficits accumulated with increased RTD were of a functional nature, although RTD at 50 meters was also associated with worse self-reported health, increased vitality problems and higher incidence of chronic morbidities. Living closer to a petrol station was associated with a higher incidence of functional impairments and chronic morbidities.

**Conclusions:**

Exposure to nearby residential traffic is associated with accelerated trajectories of unhealthy ageing. Diminishing traffic pollution should become a priority intervention for adding healthy years to life in the old age.

**Supplementary Information:**

The online version contains supplementary material available at 10.1186/s12940-024-01057-3.

## Key message

- No previous study has evaluated the influence of traffic exposure on ageing trajectories.
- Our results suggest that higher residential exposure to traffic is associated with increased functional deterioration in older adults.
- Diminishing traffic pollution should become a priority intervention for adding healthy years to life in the old age.

**Supplementary Information:**

The online version contains supplementary material available at 10.1186/s12940-024-01057-3.

## Background

One of the great challenges of the 21^st^century is global population ageing, with estimates that, by 2050, people aged 60 years and older will account for around 22% of the global population [1]. To offset the related increase in healthcare and social care costs, it is essential to identify potentially modifiable determinants of accelerated ageing, including early development of chronic diseases, functional decline, and disability. Only in this way can we design adequate public health interventions to prevent, treat or palliate the decline of health with age.

According to the Population Division of the United Nations Department of Economic and Social Affairs, around 60% of the world population will live in urban settlements by 2030. These pose special challenges for older adults, who may be more vulnerable to urban-related environmental exposures such as traffic-related air pollution (TRAP) due to a progressive decline in homeostatic mechanisms. Precisely, TRAP has been associated with biomarkers of increased biological ageing [2, 3], incidence of age-related chronic morbidities (i.e. lung cancer [4, 5], chronic obstructive pulmonary disease [5], cardiovascular disease [5, 6], hypertension [7], diabetes [5, 8], stroke [5, 9], cognitive decline [10–12], or Alzheimer's [13]), as well as increased short-term [14] and long-term [15, 16] respiratory, cardiovascular, and all-cause mortality. Traffic-related noise is another growing environmental health problem associated with urban areas, and has been linked to chronic conditions including cardiovascular disease [17], hypertension [18], diabetes mellitus [18], impaired cognitive function [19–21] and changes in brain structure [22].

Notwithstanding this vast literature, only four previous studies have assessed whether residential exposure to traffic may be associated with unhealthy ageing in older adults [23–26], two of which were of cross-sectional design [25, 26]. Moreover, none has evaluated the influence of traffic on ageing trajectories nor accounted for individual lifestyle-related factors. The present study aims to fill some of the gaps in existing research by evaluating the association between residential traffic and unhealthy ageing, as assessed through the accumulation of overall and domain-specific health deficits over a 10-year follow-up (2008-2018) of a nationally representative cohort of community-dwelling older adults in Spain. 

## Methods

### Study population and data collection

Data were taken from the Seniors-ENRICA 1 cohort, comprising 3,228 individuals recruited between 2008 and 2010 by stratified multistage random sampling of the Spanish non-institutionalized general population aged 60 years or older, and who were followed for 8,291 biannual visits. At baseline, and every 2.5 years, trained staff conducted computer-assisted telephone interviews and home visits to collect information regarding lifestyles and health-related outcomes, as well as taking blood and urine samples [27]. All participants gave written informed consent, and the study was approved by the Clinical Research Ethics Committee of the La Paz Hospital in Madrid.

### Health deficits accumulation

Based on the work by Rockwood et al., [28] we estimated unhealthy ageing with a Deficit Accumulation Index (DAI) comprising 52 items grouped into four domains: 1) 22 impairments in physical and cognitive functioning; 2) 7 self-reported health and vitality problems; 3) 6 items related to mental health deficits; and 4) 17 items on morbidities, polypharmacy and use of health services [29]. Some health deficits were assessed dichotomously (1 point if present and 0 points if not), while others were graded according to severity (0 points for no deficit, 0.25 to 0.75 points for mild to moderate deficits, and 1 point for severe deficits). The overall DAI was calculated as the sum of points assigned to each health deficit divided by the total number of deficits, multiplied by 100 to obtain a summary range from 0 to 100% deficits. Higher scores in the DAI or any of its domains indicates unhealthy ageing. A more detailed description of the construction of the DAI is provided elsewhere [29] and the complete list of health deficits and associated scores is shown in Supplementary Table 1.

### Residential traffic

The Annual Average Daily Traffic (AADT) is a tool used to predict how active a road is and it has shown to be a useful instrument when assessing the health effects of traffic density exposure [30, 31]. It is computed as the total volume of vehicle traffic on a highway, road or street for a year divided by 365 days, and is expressed in vehicles per day. AADT was measured via the Navteq cartography combined with the traffic density information provided by the Spanish Ministry of Public Works and Transport [30]. We first created buffers at 50 and 100 meters around the participantsʼ residential address home, and then, calculated residential traffic density (RTD) as the total volume of AADT within the buffers; weights were applied proportionally to the length of the road segment overlapping with the buffer. The Euclidean distance between each participant's home address and the nearest major road and gas station, registered in the Geoportal website from *the Spanish Ministry for the Ecological Transition,*were also calculated as measures of traffic exposure [32].

### Other variables

At baseline and subsequent follow-up visits, information was collected on sex, age, educational level (primary or lower, secondary, or university), alcohol consumption (never, ex-drinker, current drinkers) and tobacco smoking (never, ex-smoker, or current smoker), adherence to the Mediterranean diet (categorized as ≤ 5, 6, 7, 8, or ≥ 9 points), and average weekly hours watching TV as in the Nurses’ Health Study questionnaire validated in Spain. Recreational physical activity (expressed in METs-hour/week) was measured using the validated questionnaire from the EPIC-Spain cohort, and included walking (commuting, shopping, or leisure time), cycling (commuting or leisure time), and playing sports (running, playing soccer, doing aerobics, swimming, or playing tennis). Weight and height were measured twice using electronic scales and portable extendable stadiometers, with standardized procedures, and the mean of the two readings was used to calculate the body mass index (BMI), which was categorized as < 25, 25–29.9, or ≥ 30 kg/m^2^.

The Socioeconomic Deprivation Index (SDI), as developed by the Spanish Society of Epidemiology, was estimated based on census tract data. This index comprises six indicators: manual worker population, casual wage-earning population, unemployment level, individuals 16 and over and between 16 and 29 years of age with primary education or less, and main dwellings without internet access [33].

Green and blue spaces exposure were measured using the Spanish Land Use Information System (SIOSE) database provided by the Spanish National Geographic Institute website (IGN), which divides the terrain into areas (polygons) classifying them in different aggregation levels. As described in previous reports [34, 35], urban green (i.e. urban parks and wooden areas) and blue (i.e. natural course, lakes, natural and artificial ponds, rivers, reservoir, coastal lagoons) spaces were measured in buffers of 50 and 100 meters around the participants´ residential address.

### Statistical analyses

From the initial cohort, 79 participants were excluded because their address could not be geocoded, 60 due to lacked baseline information on health deficits or potential confounders, and 21 missing information on residential traffic density (RTD). The remaining 3,129 participants contributed updated data at 5,162 biannual follow-up visits until 2017, including 2,401, 1,680 and 1,081 participants in the first, second, and third follow-up visits, respectively (Supplementary Figure 1). The losses to follow-up were strongly related to the presence of health deficits and lifestyle determinants, as well as to residential characteristics, and the induced selection bias was corrected through weighting methods based on the inverse probability of censoring.

As explained in a previous work with this study sample [27], stabilized censoring were estimated as the probability of remaining uncensored given the corresponding exposure variable, baseline sociodemographic and lifestyle variables, SDI, and DAI, divided by that probability further conditional on lifestyle and DA histories through each visit. The probabilities of being uncensored at each follow-up visit were estimated using pooled logistic models, and the odds of remaining uncensored were estimated to decrease by 34% per 10-percent increase in DAI [27]. The distribution of censoring weights at each follow-up visit is displayed in Supplementary Figure 2.

The average difference in DAI at each visit across quintiles (Q) of the exposure variables at baseline (RTD at 50 and 100 meters, distance to petrol station, distance to major road) was estimated using marginal structural models with clustered robust standard errors to account for the repeated measures for each participant and spatial correlation at the census tract. Supplementary Figure 3 depicts the directed acyclic graph (DAG) generated in DAGitty, identifying the variables essential for adjustment in regression models. Model 1 was adjusted for age (restricted quadratic splines with knots at 65, 70, 75, and 80 years), sex, education level, quintiles of SDI and baseline DAI (restricted quadratic splines with knots at 10, 20, and 30%). Model 2 further adjusted for changes over time in smoking status, alcohol drinking, Mediterranean diet score, TV hours/day, physical activity, and BMI. Model 3 adjusted for the corresponding exposure variable (RTD in the case of distance to petrol station, and vice versa), residential green and blue spaces. Tests for linear trend in the average health deficits accumulation were performed by including an ordinal variable with the median of the quintiles of the corresponding exposure variable, and smooth dose-response curves estimated through restricted quadratic splines of exposure variables. To control for selection bias due to differential loss to follow-up with respect to time-varying DAI and residential area, stabilized censoring weights were allocated to each participant-visit. Specific effects of each exposure variable on DAI among study participants were estimated by including interactions between exposure categories and categories of sex, age, education, lifestyle factors, BMI, baseline DAI, SDI and natural spaces in repeated measures models with stabilized censoring weights. Effect modification was evaluated by Wald test.

Lastly, the prospective association of RTD at 50 and 100 metres, and distance to petrol station, with domain-specific DAI (physical and cognitive function, self-rated health and vitality, mental health, and morbidity) was estimated with similar modeling strategies to that for the overall DAI score. Statistical analyses were performed in Stata, version 17 (StataCorp).

## Results

The median (min-max) RTD at 50 and 100 meters was 47 (0-4104) and 185 (0-25283) vehicles/day, respectively, while the mean (SD) distance to the nearest petrol station and to the nearest major road was 962 (1317) and 1903 (2288) meters, respectively. No participants had a major road within the closer buffer (50 meters), and only 2.7% had a major road within 100 meters. At baseline, the mean (SD) age and DAI score of the participants were 69.0 (6.6) years and 17.0 (11.0) percent deficit, and 54.0% were women (Table 1). Participants with higher exposure to RTD showed, on average, a higher level of education, a lower deprivation index, lower exposure to blue and green spaces, a higher prevalence of tobacco and alcohol consumption, more physical activity, and a higher baseline DAI. No association between baseline individual or residential characteristics and the nearest distance to a petrol station was observed (Table 1).

**Table 1:** Baseline individual and residential characteristics by quintiles of Traffic Density on Residential streets (RTD) in a 50 and 100-meter buffer and by quintiles of residential distance to the nearest petrol station

		**RTD at 50 m**					**RTD at 100 m**					**Distance to petrol station (m)**				
		**Q1**	**Q2**	**Q3**	**Q4**	**Q5**	**Q1**	**Q2**	**Q3**	**Q4**	**Q5**	**Q1**	**Q2**	**Q3**	**Q4**	**Q5**
**Age**	69.04 (6.6)	68.84 (6.6)	68.83 (6.5)	69.19 (6.4)	69.28 (6.7)	69.05 (6.7)	68.80 (6.6)	68.39 (6.3)	69.60 (6.8)	69.41 (6.6)	69.00 (6.6)	69.08 (6.6)	69.11 (6.6)	68.86 (6.5)	69.26 (6.7)	68.89 (6.7)
**Sex**																
Male	46.05	43.44	48.73	45.13	46.09	46.79	46.81	47.31	43.55	46.33	46.31	53.96	54.18	54.08	53.17	54.35
Female	53.95	56.56	51.27	54.87	53.91	53.21	53.19	52.69	56.45	53.67	53.69	46.04	45.82	45.92	46.83	45.65
**Education**																
≤Primary	57.14	62.46	58.23	60.53	53.91	50.64	61.54	58.07	58.81	54.15	53.21	56.80	59.49	56.32	57.30	55.81
Secondary	23.68	20.66	24.21	23.27	24.72	25.48	21.93	23.73	23.27	23.81	25.64	23.89	22.67	23.20	23.49	25.16
University	19.18	16.89	17.56	16.19	21.37	23.88	16.53	18.20	17.92	22.04	21.15	19.30	17.85	20.48	19.21	19.03
**Deprivation index**	-0.32 (0.9)	-0.02 (0.9)	-0.40 (0.8)	-0.17 (0.9)	-0.50 (0.9)	-0.51 (0.8)	-0.15 (0.9)	-0.24 (0.8)	-0.24 (1.0)	-0.38 (0.8)	-0.61 (0.8)	-0.33 (0.9)	-0.28 (0.9)	-0.30 (0.9)	-0.34 (0.8)	-0.37 (0.9)
**Smoking**																
Never	58.20	61.15	58.70	58.33	58.21	54.65	60.72	56.81	59.59	57.99	55.93	55.38	59.81	59.04	58.41	58.39
Former	30.20	27.05	29.27	30.82	31.10	32.69	26.19	31.01	31.13	31.15	31.41	31.80	29.26	30.88	30.63	28.39
Current	11.60	11.80	12.03	10.85	10.69	12.66	13.09	12.18	9.28	10.86	12.66	12.82	10.93	10.08	10.95	13.23
**Alcohol intake**																
Never	30.90	34.75	26.74	31.29	33.33	28.53	33.72	28.96	31.45	32.91	27.56	30.22	33.28	33.12	27.30	30.65
Former	54.59	52.79	59.34	55.19	51.20	54.33	54.66	57.91	54.09	51.76	54.49	53.16	55.14	52.32	56.98	55.32
Current	14.51	12.46	13.92	13.52	15.47	17.15	11.62	13.13	14.47	15.34	17.95	16.61	11.58	14.56	15.71	14.03
**MDS**	6.87 (1.8)	6.91 (1.8)	6.81 (1.8)	6.81 (1.8)	7.00 (1.7)	6.81 (1.8)	6.94 (1.7)	6.86 (1.9)	6.78 (1.7)	6.87 (1.8)	6.88 (1.7)	6.87 (1.7)	6.91 (1.7)	6.85 (1.8)	6.88 (1.8)	6.82 (1.8)
**BMI**	28.54 (4.4)	28.84 (4.6)	28.56 (4.2)	28.67 (4.5)	28.31 (4.3)	28.33 (4.4)	28.65 (4.4)	28.68 (4.5)	28.60 (4.4)	28.42 (4.3)	28.35 (4.4)	28.56 (4.1)	28.38 (4.5)	28.44 (4.6)	28.76 (4.3)	28.56 (4.5)
**Physical activity** (MET-hours/wk)	21.47 (15.2)	19.50 (14.0)	22.28 (15.4)	20.77 (15.0)	22.48 (15.7)	22.28 (15.4)	20.10 (14.7)	21.62 (14.8)	20.64 (15.2)	21.99 (14.6)	22.99 (16.2)	20.47 (14.7)	21.35 (14.8)	22.29 (16.0)	21.98 (14.7)	21.27 (15.4)
**TV watching** (h/d)	2.64 (1.6)	2.71 (1.6)	2.83 (1.7)	2.68 (1.6)	2.48 (1.6)	2.48 (1.7)	2.71 (1.7)	2.70 (1.6)	2.69 (1.7)	2.54 (1.6)	2.55 (1.7)	2.62 (1.6)	2.68 (1.7)	2.68 (1.7)	2.63 (1.6)	2.57 (1.6)
**Baseline DAI**	17.00 (11.0)	16.48 (10.1)	16.85 (11.3)	17.10 (11.1)	17.37 (11.0)	17.17 (11.6)	16.47 (10.4)	16.93 (11.3)	17.31 (11.0)	16.89 (10.7)	17.37 (11.7)	17.64 (11.1)	17.18 (10.8)	16.96 (11.2)	16.90 (11.0)	16.30 (10.9)
**Tree canopy at 500 m**	1.47 (0.9)	1.15 (0.8)	1.57 (0.7)	1.37 (0.8)	1.58 (0.9)	1.64 (0.9)	1.24 (0.8)	1.52 (0.8)	1.35 (0.9)	1.55 (0.9)	1.65 (0.9)	1.57 (1.0)	1.57 (1.0)	1.55 (1.0)	1.57 (1.0)	1.62 (1.2)
**% blue spaces at 50 m**	0.07 (0.1)	0.09 (0.2)	0.08 (0.1)	0.08 (0.1)	0.07 (0.1)	0.05 (0.1)	1.00 (0.2)	0.09 (0.1)	0.07 (0.1)	0.06 (0.1)	0.06 (0.1)	0.07 (0.1)	0.08 (0.1)	0.07 (0.1)	0.07 (0.1)	0.08 (0.1)

*RTD* Residential Traffic Density, *m* meters, *Q* Quintiles, *MDS* Mediterranean Diet Score, *BMI* Body mass index, *wk* week, *h/d* hours/day, *DAI* Deficits Accumulation Index

Data correspond to mean values (Standard Deviation) for continuous variables and percentages for categorical variables

In models weighted to control for selection bias due to differential loss to follow-up, and fully adjusted for potential confounders, a positive association of RTD and DAI was observed (Table 2, model 3). An interquartile range increase in RTD at 50 and 100 meters was associated with mean increases in DAI score of 0.71 (95%confidence interval: 0.16, 1.25), and 0.83 (0.02, 1.64) deficits, respectively. By domains, most of the deficits accumulated with increased RTD were of a *functional* nature, although RTD at 500 meters was also associated with worse self-reported health and increased vitality problems. Living closer to a petrol station was associated with a higher incidence of *functional impairments* and *chronic morbidities*.

**Table 2:** Differences^d^in health deficits accumulation by traffic density on residential streets (RTD), residential distance to the nearest petrol station and residential distance to a major road among participants in the Seniors-ENRICA cohort: 2008-2010 to 2017

**Exposure variables**	**Model 1**^**a**^	**Model 2**^**b**^	**Model 3**^**c**^
**RTD in a 50 m buffer (vehicles/day)**			
**Q1 (<22)**	Ref.	Ref.	Ref.
**Q2 (22-58)**	0.18 (-0.33, 0.69)	0.28 (-0.22, 0.78)	1.51 ( 0.50, 2.53)
**Q3 (59-189)**	0.22 (-0.32, 0.75)	0.28 (-0.24, 0.80)	0.98 (-0.05, 2.01)
**Q4 (189-592)**	0.49 (-0.04, 1.02)	**0.64 ( 0.13, 1.16)**	**2.20 ( 1.18, 3.21)**
**Q5 (≥592)**	**0.53 ( 0.00, 1.07)**	**0.67 ( 0.14, 1.20)**	**1.98 ( 0.90, 3.05)**
**p-trend**^**e**^	**0.046**	**0.014**	**0.002**
**Per-iqr**	**0.58 (0.63, 1.13)**	**0.69 (0.15, 1.23)**	**0.71 (0.16,1.25)**
**RTD in a 100 m buffer (vehicles/day)**			
**Q1 (<145)**	Ref.	Ref.	Ref.
**Q2 (146-286)**	0.03 (-0.49, 0.54)	0.04 (-0.45, 0.54)	0.04 (-0.47, 0.55)
**Q3 (287-1262)**	-0.13 (-0.66, 0.40)	-0.12 (-0.65, 0.40)	-0.08 (-0.62, 0.46)
**Q4 (1262-2144)**	**0.67 ( 0.14, 1.19)**	**0.71 ( 0.20, 1.22)**	**0.67 ( 0.14, 1.19)**
**Q5 (≥2145)**	0.17 (-0.39, 0.73)	0.23 (-0.32, 0.79)	0.27 (-0.29, 0.84)
**p-trend**^**e**^	0.228	0.137	0.137
**Per-iqr**	0.64 (-0.17, 1.44)	**0.70 (0.01, 1.58)**	**0.783 (0.02, 1.64)**
**Distance to petrol station (meters)**			
**Q1 (<365)**	Ref.	Ref.	Ref.
**Q2 (365-540)**	**-0.64 (-1.21, -0.07)**	**-0.60 (-1.16, -0.04)**	**-0.57 (-1.14, -0.01)**
**Q3 (541-750)**	**-0.74 (-1.31, -0.17)**	**-0.67 (-1.22, -0.02)**	**-0.66 (-1.21, -0.11)**
**Q4 (751-1114)**	-0.50 ( -1.07, 0.07)	-0.45 ( -1.00, 0.11)	-0.43 ( -0.99, 0.13)
**Q5 (≥1115)**	**-0.92 (-1.47, -0.38)**	**-0.92 (-1.45, -0.40)**	**-0.91 (-1.44, -0.39)**
**p-trend**^**e**^	**0.008**	**0.004**	**0.005**
**Per-iqr**	**-1.66 (-3.13, -0.19)**	**-1.81 (-3.21, -0.41)**	**-1.80 (-3.21, -0.40)**
**Distance to major road (meters)**			
**Q1 (<544)**	Ref.	Ref.	Ref.
**Q2 (545-993)**	**-0.51 (-1.01, -0.01)**	-0.43 (-0.92, 0.07)	-0.43 (-0.92, 0.06)
**Q3 (994-1552)**	-0.23 (-0.75, 0.28)	-0.18 (-0.69, 0.33)	-0.19 (-0.70, 0.32)
**Q4 (1553-2460)**	0.08 (-0.45, 0.62)	0.16 (-0.36, 0.68)	0.16 (-0.36, 0.68)
**Q5 (≥2461)**	-0.28 (-0.80, 0.24)	-0.29 (-0.81, 0.23)	-0.26 (-0.78, 0.26)
**p-trend**^**e**^	0.883	0.747	0.740
	0.27 (-0.81, 1.36)	0.29 (-0.78, 1.37)	0.28 (-0.82, 1.38)

^**a**^Model 1 was adjusted for age (restricted quadratic splines with knots at 65, 70, 75, and 80 years), sex (men or women), educational level (primary or less, secondary, or university), SDI, and baseline levels of deficits accumulation index (restricted quadratic splines with knots at 10, 20, and 30%).

^**b**^Model 2 was further adjusted for baseline and current lifestyle variables: smoking status (never, former, or current), alcohol drinking (never, former, or current), Mediterranean diet score (≤ 5, 6, 7, 8, or ≥ 9 points), body mass index (< 25, 25–29.9, or ≥ 30 kg/m2), recreational physical activity (METS-h/week), and television viewing time (hours/day).)

^**c**^Model 3 was further adjusted for the corresponding exposure variable (RTD in the case of distance to petrol station, and vice versa), for residential tree canopy and percentage of artificial blue spaces within the corresponding buffer (50 or 100 meters)

^**d**^Average differences in deficits accumulation index at each follow-up visit and 95% confidence intervals (CIs) by category of traffic exposure variable

^**e**^Tests for linear trend included an ordinal variable with the median of the corresponding quintile for each exposure variable

Regarding the nearest distance to a petrol station and prospective changes in DAI, an inverse dose-response association was observed: results in quintiles Q2 to Q5 versus Q1 were -0.57 (-1.14, -0.01), -0.66 (-1.21, -0.11), -0.43 (-0.99, 0.13), and -0.91 (-1.44, -0.39), respectively (*P* for linear trend=0.005) (Table 2, model 3). This decreased deficit accumulation with distance was particularly marked for the domains of *functional impairments* and *morbidities and use of health services:* participants living the farthest from petrol stations showed an average 1.30 (-2.31, -0.29) and 0.90 (-1.52, -0.17) lower increase in each domain over time, respectively (Table 3). Finally, no association was observed for residential distance to a major road and the accumulation of health deficits over time (Table 2).

**Table 3:** Differences in the accumulation of impairments in the four dimensions of the deficits accumulation index by traffic density on residential streets (RTD) and residential nearest distance to a petrol station, among participants in the Seniors-ENRICA cohort: 2008-2010 to 2017

	**Functional impairments**	**Self-reported health and vitality problems**	**Mental health impairments**	**Morbidities and use of health services**
**RTD in a 50 m buffer**				
**Q1**	Ref.	Ref.	Ref.	Ref.
**Q2**	0.13 (-0.84, 1.10)	0.09 (-1.05, 1.22)	0.55 (-0.76, 1.87)	0.51 (-0.23, 1.25)
**Q3**	0.60 (-0.43, 1.63)	0.43 (-0.67, 1.53)	0.20 (-1.54, 1.14)	0.35 (-0.40, 1.10)
**Q4**	**1.07 ( 0.07, 2.07)**	0.80 (-0.31, 1.92)	0.73 (-0.71, 2.18)	0.30 (-0.50, 1.09)
**Q5**	**1.15 ( 0.13, 2.17)**	1.06 (-0.05, 2.27)	0.14(-1.27, 1.55)	0.47 (-0.28, 1.22)
**p-trend**^a^	**0.025**	0.071	0.881	0.467
**RTD in a 100 m buffer**				
**Q1**	Ref.	Ref.	Ref.	Ref.
**Q2**	-0.07 (-1.02, 0.88)	-0.27 (-1.36, 0.82)	0.05 (-1.32, 1.43)	0.21 (-0.55, 0.96)
**Q3**	-0.16 (-1.20, 0.88)	-0.08 (-1.21, 1.05)	-0.16 (-1.63, 1.32)	-0.08 (-0.86, 0.70)
**Q4**	0.85 (-0.15, 1.85)	**1.19 ( 0.07, 2.31)**	0.67 (-0.85, 2.20)	0.39 (-0.42, 1.19)
**Q5**	0.58 (-0.41, 1.56)	0.38 (-0.82, 1.57**)**	-0.59 (-2.09, 0.91)	-0.08 (-0.89, 0.72)
**p-trend**^a^	0.064	0.182	0.531	0.967
**Distance to petrol station**				
**Q1**	Ref.	Ref.	Ref.	Ref.
**Q2**	-0.59 (-1.67, 0.50)	-0.60 (-1.76, 0.56)	-0.35 (-1.74, 1.05)	-0.59 (-1.38, 0.21)
**Q3**	-0.98 (-2.02, 0.06)	-0.64 (-1.82, 0.53)	**-1.35 (-2.70, -0.01)**	-0.27 (-1.00, 0.47)
**Q4**	-0.30 (-1.41, 0.81)	-0.50 (-1.66, 0.66)	-1.16 (-2.53, 0.21)	-0.23 (-0.98, 0.51)
**Q5**	**-1.30 (-2.31, -0.29)**	-0.75 (-1.92, 0.42)	-0.15 (-1.56, 1.27)	**-0.90 (-1.52, -0.17)**
**p-trend**^a^	**0.024**	0.339	0.893	**0.037**

Models were adjusted for individual sociodemographic variables, level, SDI, baseline deficits in each dimension, baseline and current lifestyle variables, RTD or distance to petrol station (as corresponding), residential tree canopy and percentage of artificial blue spaces within 50 meters

^a^Tests for linear trend included an ordinal variable with the median of the corresponding quintile for each exposure variable

**Fig. 1:**
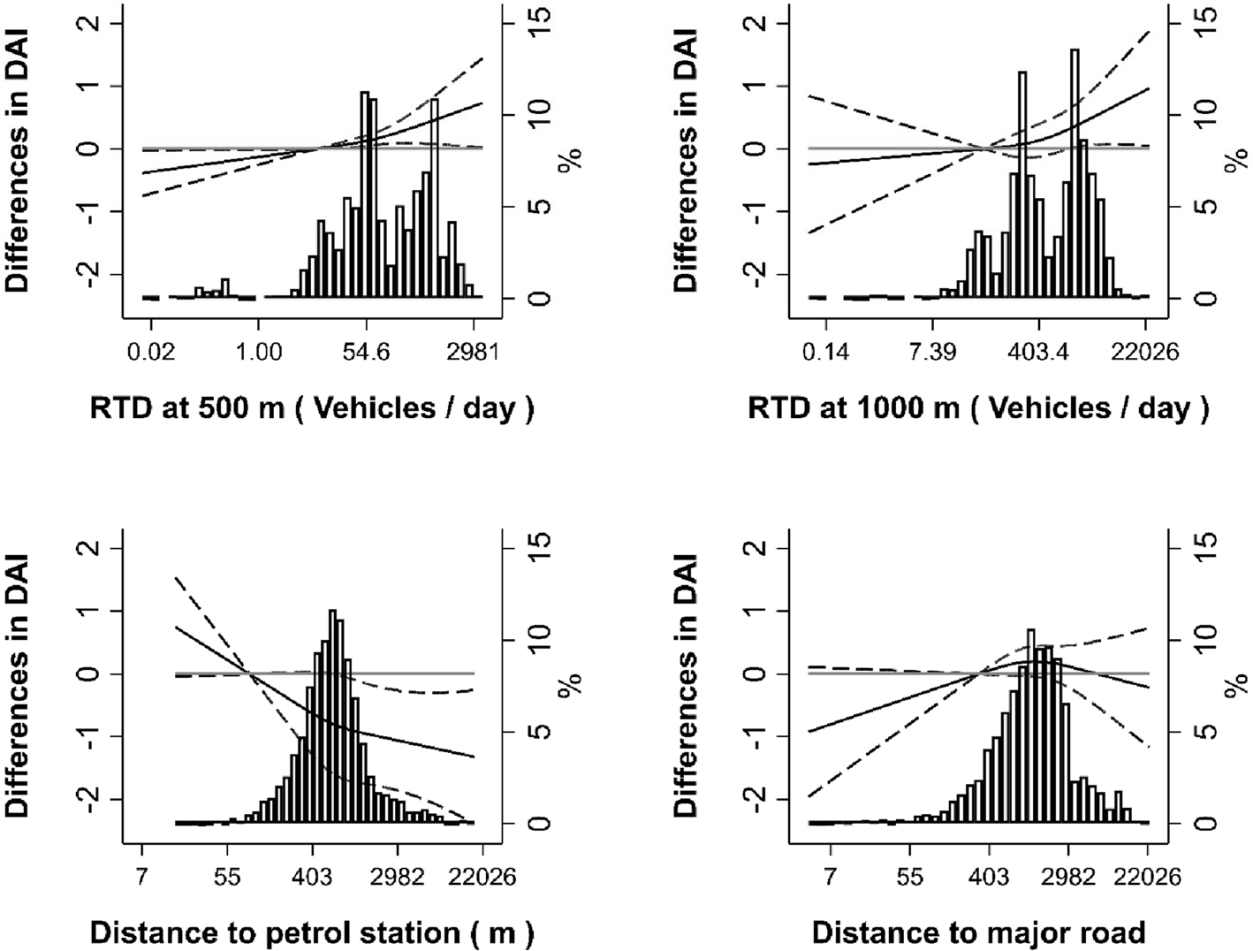
Differences in health deficits accumulation as a smooth function of residential traffic density (RTD) at 50 and 100 meters and of residential nearest distance to a petrol station or a major road in the Seniors-ENRICA cohort, 2008–2010 to 2017. Curves represent average differences in deficits accumulation index at each follow-up visit (solid lines) and 95% confidence intervals (dashed lines) based on restricted cubic splines for the exposure variables at baseline. The reference values were set at the 10 th percentile of each exposure variable distribution (8.8 vehicles/day, 32.4 vehicles/day, 254.8 meters and 310 meters, respectively). Average differences were obtained from repeated measures regression models adjusted for age, sex, educational level, baseline levels of the deficit accumulation index (DAI), time-varying lifestyles, social deprivation index (SDI) at the census tract, and presence of natural spaces. Models were weighted by the inverse of the conditional probabilities of censoring given residential exposure to traffic variables, individual time-varying confounders, SDI and presence of natural spaces; and accounted for spatial correlation at the census tract and within-participant correlations induced by repeated measures and weighting. Bars represent the histograms of the exposure variables

Smooth dose-response analyses with reference values set at the 10^th^ percentile of each exposure variable showed non-significant departures from linearity (*P* non-linear trend for RTD at 50, RTD at 100 m, and distance to petrol station= 0.39, 0.35, and 0.41, respectively) (Fig. 1).

In exploratory subgroup analyses, the average deleterious changes in DAI with increasing exposure to traffic-related variables was similar across participants´ characteristics (Supplementary Figure 3).

## Discussion

Our results suggest that increased exposure to nearby residential traffic is associated with increased health deficit accumulation, particularly with functional deterioration, in older adults.

Up to date, only four previous studies have evaluated the association between TRAP and unhealthy ageing, and none of the longitudinal reports have repeated measures over time. In the first study, conducted with 848 individuals aged ≤65 hospitalized for incident myocardial infarction in 1992 and 1993 in central Israel, baseline exposure to higher concentrations of PM2.5 (as estimated from eight 24-hour air quality monitoring stations distributed throughout central Israel) was associated with an increased risk of developing DAI scores ≥0.25 [23]. In the second, authors used longitudinal data from 6570 adults aged ≥65 from the Chinese Longitudinal Healthy Longevity Survey linked with aggregated air quality data for 117 cities in China and observed that participants living in cities with very unhealthy air quality compared to those with good air quality (as registered one year before the first interview), had a 23% higher increase in DAI over a 3-year period. Moreover, old people living in areas where air pollution increased over the follow-up had larger increases in DAI scores than those where air pollution was relatively constant [24]. Unfortunately, though, the authors did not account for individual lifestyle risk factors into the analyses, did not provide validated measures of functionality other than self-report, and did not evaluate health domain-specific associations. The third study, evaluated the cross-sectional association between daily average concentrations of PM_2.5_, PM_10_and ozone measured at 268 nationwide surveillance stations in South Korea and matched with the residential address of 2912 community-dwelling older adults, with the prevalence of frailty assessed with a modified version of the Korean frailty scale [25]. The authors found that for each 1 µg/m^3^ increase in PM_2.5_ and PM_10_, the prevalence of frailty increased by about 0.5%. Finally, the most recent study on this topic, showed a cross-sectional association between levels of PM_2.5_registered 1 to 5 years prior to the interview and prevalence of DAI scores ≥0.20 in 34,138 participants from 6 low and middle-income countries [26]. Data on PM_2.5_ were derived from a combination of observations from the Moderate Resolution Imaging Spectroradiometer and Multiangle Imaging Spectroradiometer instruments from the Terra Satellite, along with simulations from the GEOS-Chem chemical transport model. The study adjusted for individual lifestyle-related risk factors and found that, in rural but not urban areas, each 10 µg/m^3^ increase in PM_2.5_was associated with a 30% increase in the odds of having DAI scores ≥20 [19].

Several mechanisms can explain why residential traffic exposure may influence physical and cognitive functions in older adults. First, ageing is characterized by chronic low-grade inflammation [36] and a compromised antioxidant response [37], with strong evidence that exposure to ambient particulate air pollution is associated with increased circulating levels of IL-6 [38, 39] and C-reactive protein [38–40], increased stimulated production of inflammatory cytokines (i.e. IL-6, IL-1β, and TNF-α) [41], and increased oxidative stress biomarkers (i.e. malondialdehyde, 8-hydroxy-2'-deoxyguanosine or superoxide dismutase) [42]. In experimental animal models, traffic noise has also been linked to increased oxidative stress and inflammation [43]. Second, ageing is associated with lower IGF-1 and increased insulin resistance, and exposure to air pollutants has shown to be a risk factor of insulin resistance [44] and reduced insulin sensitivity over time [45]. Third, there is growing evidence of brain-structure neurotoxicity associated with PM [46–48] and road traffic noise exposures [22]. Fourth, genome-wide DNA methylation analyses suggest that exposure to long-term ambient air pollution can lead to alterations in DNA methylation whose functions are related to mitochondria and immune responses [49]. Fifth, air pollution has been associated with accelerated biological ageing as assessed using telomere length and DNA methylation ageing clocks [3, 50]. And finally, exposure to air pollutants and noise can exacerbate numerous chronic conditions including cardiovascular or metabolic processes, increasing the risk of functional decline.

Our study has important strengths. Not only is it the first to evaluate the association between exposure to residential traffic and ageing trajectories, but it is also the first to address the link between residential traffic and functional ageing while controlling for time-varying individual lifestyle related factors. Also, the study uses a nationally representative sample of community-dwelling older adults and focuses on exposure contrasts at the neighborhood scale (vs urban or regional scale), which has been shown to offer the greatest potential in determining associations with outcomes derived from traffic pollution [16]. Among the limitations, we used RDT and distance to petrol stations as proxies for residential exposure to urban traffic instead of quantifying the exposure to each traffic-related air pollutant (i.e. , particulate matter, nitrogen dioxide, carbon monoxide, aldehydes, benzene, sulfur dioxide, polycyclic aromatic hydrocarbons etc.) or to traffic-related noise, and further research in needed to evaluate which traffic components mediate the observed associations.

## Conclusions

Our results suggest that traffic pollution may accelerate trajectories of unhealthy ageing. Given the increasing life expectancy of the population and the widespread exposure to high traffic density, diminishing traffic pollution should become a priority intervention for adding healthy years to life in the old age.

### Supplementary Information

**Additional file 1: Supplementary Table 1.** Health deficits and associated scores in the deficits accumulation index.** Supplementary Figure 1.** Study flow diagram.** Supplementary Figure 2.** Distributions of stabilized censoring weights by follow-up visit in the Seniors-ENRICA cohort, 2008–2010 to 2017.** Supplementary Figure 3.** Proposed directed acyclic graph for the relationship between residential traffic exposure metabolites and the accumulation of health deficits.** Supplementary Figure 4.** Differences in health deficits accumulation by traffic-related exposure variables in subgroups of participants in the Seniors-ENRICA cohort, 2008–2010 to 2017. Subgroup-specific average differences in deficits accumulation index at each follow-up visit and 95% confidence intervals (CIs,horizontal lines) by category of exposure variables were obtained from repeated measures regression models with interactions between the exposures (expresed as interquartile range) and the corresponding subgroups; with clustered robust standard error to account for the repeated measures for each participant and spatial correlation at the census tract; adjusted for age, sex, education, smoking status, alcohol drinking, Mediterranean diet score, body mass index, recreational physical activity, sedentary behavior, baseline levels of deficits accumulation index, Social Deprivation Index at the census tract, and residential exposure to natural spaces; weighted by the inverse of the conditional probabilities of censoring given follow-up levels of the above factors.

## Data Availability

The datasets used and/or analysed during the current study are available from the corresponding author on reasonable request.

## Abbreviations

AADT: Annual Average Daily Traffic
BMI: Body Mass Index
DAI: Deficit Accumulation-Index
IQR: Interquartile Range
RTD: Residential traffic density
SDI: Socioeconomic Deprivation Index
TRAP: Traffic-related air pollution
