# Supplementary material for: Exposure to residential traffic and trajectories of unhealthy ageing: results from a nationally-representative cohort of older adults
Source: Environ Health. 2024 Feb 1;23:15. doi: 10.1186/s12940-024-01057-3 (PMC10832178; doi:10.1186/s12940-024-01057-3)
Supplement: Supplementary file 1 — Additional file 1: Supplementary Table 1. Health deficits and associated scores in the deficits accumulation index. Supplementary Figure 1. Study flow diagram. Supplementary Figure 2. Distributions of stabilized censoring weights by follow-up visit in the Seniors-ENRICA cohort, 2008–2010 to 2017. Supplementary Figure 3. Proposed directed acyclic graph for the relationship between residential traffic exposure metabolites and the accumulation of health deficits. Supplementary Figure 4. Differences in health deficits accumulation by traffic-related exposure variables in subgroups of participants in the Seniors-ENRICA cohort, 2008–2010 to 2017. Subgroup-specific average differences in deficits accumulation index at each follow-up visit and 95% confidence intervals (CIs,horizontal lines) by category of exposure variables were obtained from repeated measures regression models with interactions between the exposures (expresed as interquartile range) and the corresponding subgroups; with clustered robust standard error to account for the repeated measures for each participant and spatial correlation at the census tract; adjusted for age, sex, education, smoking status, alcohol drinking, Mediterranean diet score, body mass index, recreational physical activity, sedentary behavior, baseline levels of deficits accumulation index, Social Deprivation Index at the census tract, and residential exposure to natural spaces; weighted by the inverse of the conditional probabilities of censoring given follow-up levels of the above factors. [file 12940_2024_1057_MOESM1_ESM.docx]

**Supplementary Table 1.** Health deficits and associated scores in the deficits accumulation index.

| **Health deficit** | **Score** |
| --- | --- |
| **Physical and cognitive impairments** |  |
| *Self-care disability* |  |
| Help bathing | no=0, yes=1 |
| Help eating | no=0, yes=1 |
| Help dressing | no=0, yes=1 |
| Help using the toilet | no=0, yes=1 |
| Incontinence | no=0, yes=1 |
| *Disability in instrumental activities of daily living* |  |
| Help shopping | no=0, yes=1 |
| Help preparing meals | no=0, yes=1 |
| Help with housework | no=0, yes=1 |
| Help taking medications | no=0, yes=1 |
| Help with finances | no=0, yes=1 |
| *Agility disability* |  |
| Limitation in bending or kneeling | no=0, yes=1 |
| *Mobility disability* |  |
| Limitation to lift/carry a shopping bag | no=0, yes=1 |
| Limitation in climbing one flight of stairs | no=0, yes=1 |
| Limitation to walk several blocks | no=0, yes=1 |
| Limitation in moderate activities | no=0, yes=1 |
| Decreased life-space mobility | no=0, yes=1 |
| *Limitation in lower-extremity physical performance* |  |
| Poor balance | no=0, yes=1 |
| Slowness | no=0, yes=1 |
| Unable to complete five chair stands | no=0, yes=1 |
| *Low strength* |  |
| Lowest grip strength quintile according to sex and BMI | no=0, yes=1 |
| *Cognitive impairment* |  |
| Mini-Mental State Examination score | ≥24=0, 20–23=0.25, 18–19=0.5, 11–17=0.75, ≤10=1 |
| **Self-rated health and vitality** |  |
| Self-rated health | excellent=0, very good=0.25, good=0.5, fair=0.75, poor=1 |
| Doing less as a result of physical health | never=0, rarely=0.25, sometimes=0.5, mostly=0.75, always=1 |
| Limited in activities as a result of physical health | never=0, rarely=0.25, sometimes=0.5, mostly=0.75, always=1 |
| Health interfered with social activities | never=0, rarely=0.25, sometimes=0.5, mostly=0.75, always=1 |
| Pain interfered with normal work | never=0, rarely=0.25, sometimes=0.5, mostly=0.75, always=1 |
| Not having energy | never=0, rarely=0.25, sometimes=0.5, mostly=0.75, always=1 |
| Unintentional weight loss | no=0, yes=1 |

(Table continues)

**Supplementary Table 1** (continued).

| **Health deficit** | **Score** |
| --- | --- |
| **Mental health** |  |
| Accomplishing less because of emotional problems | never=0, rarely=0.25, sometimes=0.5, mostly=0.75, always=1 |
| Doing activities less carefully than usual | never=0, rarely=0.25, sometimes=0.5, mostly=0.75, always=1 |
| Not feeling calm and peaceful | never=0, rarely=0.25, sometimes=0.5, mostly=0.75, always=1 |
| Feeling down-hearted and blue | never=0, rarely=0.25, sometimes=0.5, mostly=0.75, always=1 |
| Not being able to face up to problems | no=0, yes=1 |
| Feeling helpless | no=0, yes=1 |
| **Morbidities, polypharmacy, and use of health services** |  |
| *Morbidities* |  |
| Heart attack | no=0, yes=1 |
| Heart failure | no=0, yes=1 |
| Stroke | no=0, yes=1 |
| Cancer | no=0, yes=1 |
| Respiratory disease | no=0, yes=1 |
| Arthritis | no=0, yes=1 |
| Osteoarthritis | no=0, yes=1 |
| Hip fracture | no=0, yes=1 |
| Parkinson’s disease | no=0, yes=1 |
| Periodontal disease | no=0, yes=1 |
| Diabetes | no=0, yes=1 |
| Depression | no=0, yes=1 |
| Underweight/severe obesity (body mass index) | 18.5–<35=0, <18.5 or ≥35=1 |
| Hypertension | no=0, yes=1 |
| *Polypharmacy* |  |
| No. of medications | 0–2=0, 3–4=0.5, ≥5=1 |
| *Use of health services* |  |
| Frequency of outpatient health care in last 12 months | rarely=0, once/twice a year=0.25, every two/three months=0.5, once/twice a month=0.75, almost every day=1 |
| Overnight stay in hospital in last 12 months | no=0, yes=1 |

**Supplementary Figure 1. Study flow diagram.**

**Initial cohort** (n = 3,289)

**Excluded** (n=160)

Address could not be geocoded (n=79)

Missing information on traffic density (n=21)

Missing information on health deficits or potential confounders (n=60)

**2008-2010 baseline visit** (n =3,129)

**Lost to follow-up** (n=728)

Death (n =96)

Non-response (n =632)

**2013 first follow-up visit** (n =2,401)

**Lost to follow-up** (n=721)

Death (n =172 )

Non-response (n =549)

**2015 second follow-up visit** (n =1,680)

**Lost to follow-up** (n=599)

Death (n =339 )

Non-response (n =260)

**2017 third follow-up visit** (n =1,081)

**Analyzed** (**8,291** baseline and follow-up visits)

**Supplementary Figure 2.** Distributions of stabilized censoring weights by follow-up visit in the Seniors-ENRICA cohort, 2008–2010 to 2017.


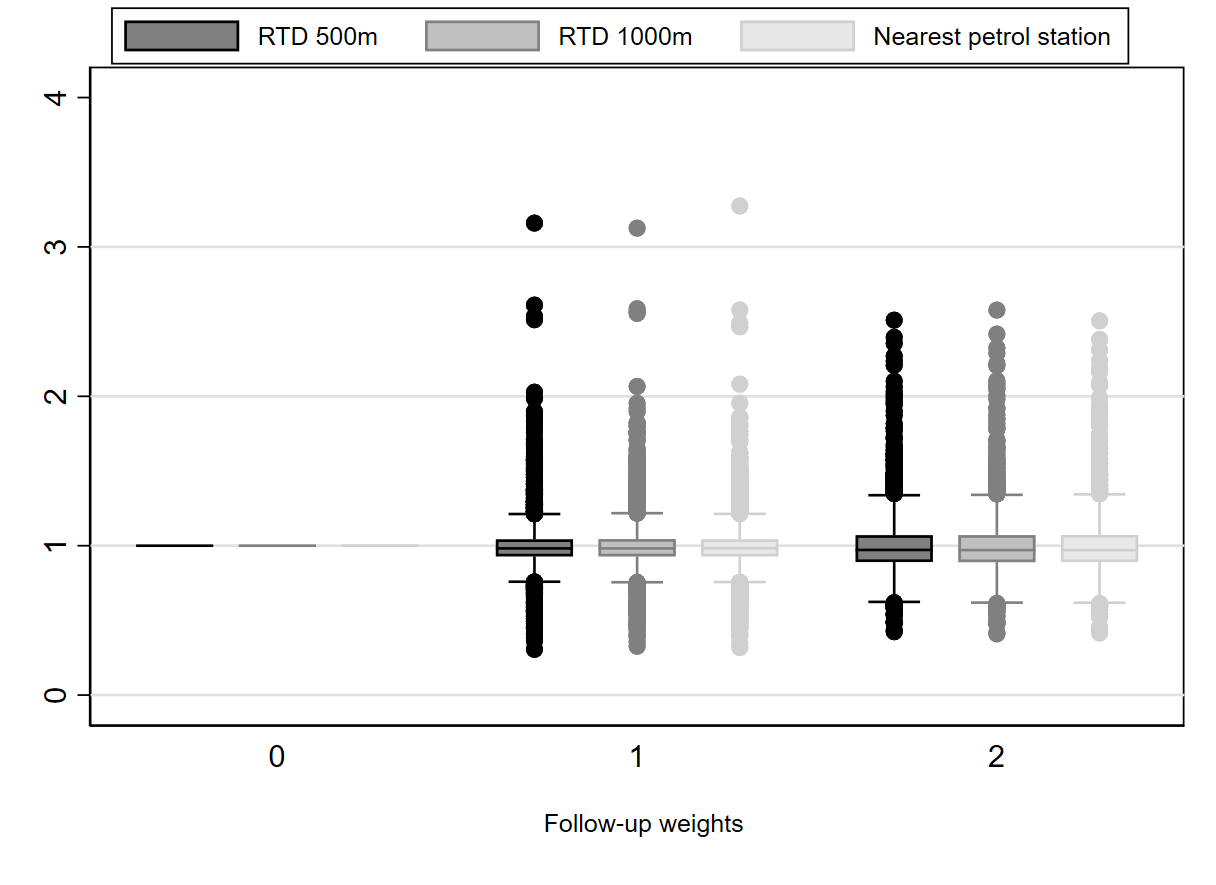


Weights were set at 1 at the first follow-up visit and the boxes for the second and third follow-up visits are shown.

**Supplementary Figure 3.** Proposed directed acyclic graph for the relationship between residential traffic exposure metabolites and the accumulation of health deficits.

**
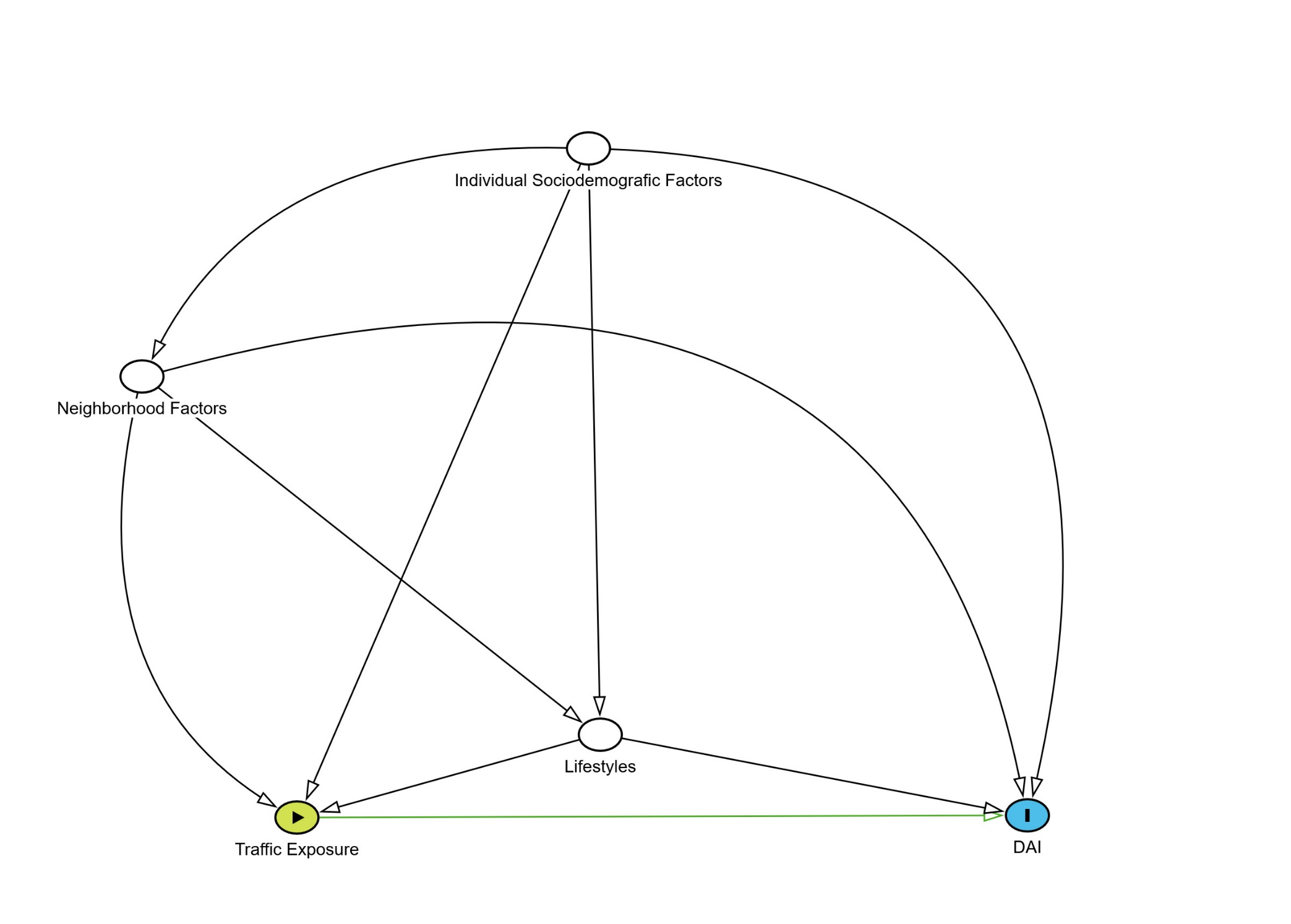
**

**Supplementary Figure 4**. Differences in health deficits accumulation by traffic-related exposure variables in subgroups of participants in the Seniors-ENRICA cohort, 2008–2010 to 2017. Subgroup-specific average differences in deficits accumulation index at each follow-up visit and 95% confidence intervals (CIs,horizontal lines) by category of exposure variables were obtained from repeated measures regression models with interactions between the exposures (expresed as interquartile range) and the corresponding subgroups; with clustered robust standard error to account for the repeated measures for each participant and spatial correlation at the census tract; adjusted for age, sex, education, smoking status, alcohol drinking, Mediterranean diet score, body mass index, recreational physical activity, sedentary behavior, baseline levels of deficits accumulation index, Social Deprivation Index at the census tract, and residential exposure to natural spaces; weighted by the inverse of the conditional probabilities of censoring given follow-up levels of the above factors.

**
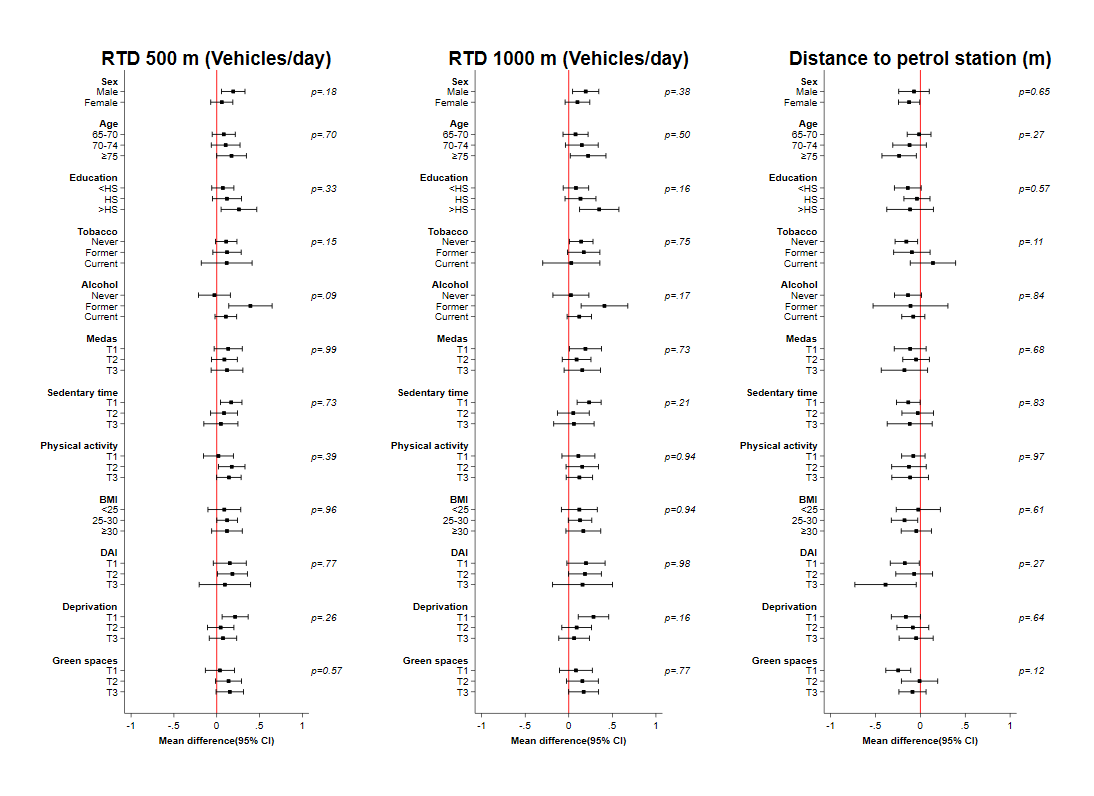
**

T1-T3: First to third tertiles; Adherence to Mediterranean Diet Score; BMI: Body Mass Index; DAI: Deficits Accumulation Index; Deprivation: Social Deprivation Index
